# Impact of lenvatinib on renal function compared to sorafenib for unresectable hepatocellular carcinoma

**DOI:** 10.1097/MD.0000000000029289

**Published:** 2022-05-20

**Authors:** Ryu Sasaki, Masanori Fukushima, Masafumi Haraguchi, Takuya Honda, Satoshi Miuma, Hisamitsu Miyaaki, Kazuhiko Nakao

**Affiliations:** Department of Gastroenterology and Hepatology, Nagasaki University Graduate School of Biomedical Sciences, 1-7-1 Sakamoto, Nagasaki City, Nagasaki, Japan.

**Keywords:** estimated glomerular filtration rate, hepatocellular carcinoma, lenvatinib, proteinuria, renal function, sorafenib, tyrosine kinase inhibitors

## Abstract

Anti-VEGF drugs, such as tyrosine kinase inhibitors, play an important role in systemic therapy for unresectable hepatocellular carcinoma (uHCC). We examined the effects of sorafenib and lenvatinib on proteinuria and renal function.

Patients who were administered sorafenib (n = 85) or lenvatinib (n = 52) as first line treatment for uHCC from July 2009 to October 2020, were enrolled in this retrospective observational study. A propensity score analysis including 13 baseline characteristics was performed. Eighty four patients were selected (sorafenib, n = 42; lenvatinib, n = 42) by propensity score matching (one-to-one nearest neighbor matching within a caliper of 0.2). We analyzed changes in estimated glomerular filtration rate (eGFR) during tyrosine kinase inhibitor treatment, as well as the development of proteinuria in both groups. A multivariate analysis was performed to identify predictors of a deterioration of eGFR.

At 4, 8, 12, and 16 weeks, ΔeGFR was significantly lower in the lenvatinib group than in the sorafenib group (*P* < .05). The lenvatinib group showed a significantly higher frequency of proteinuria than the sorafenib group (30.9% vs 7.1%, *P* = .005) and had a higher rate of decrease in eGFR than the sorafenib group (*P* < .05). Multivariate analysis revealed that lenvatinib use was the only predictive factor of eGFR deterioration (odds ratio 2.547 [95% CI 1.028–6.315], *P* = .043). In cases of proteinuria ≤1+ during lenvatinib treatment, eGFR did not decrease. However, eGFR decreased in the long term (>24 weeks) in patients who have proteinuria ≥2+.

Lenvatinib has a greater effect on proteinuria and renal function than sorafenib. In performing multi-molecular targeted agent sequential therapy for uHCC, proteinuria and renal function are important factors associated with drug selection after atezolizumab-bevacizumab combination therapy currently used as the first-line treatment.

## 1 Introduction

Angiogenesis plays a vital role in the pathogenesis of malignancies through the production of pro-angiogenic molecules, of which vascular epidermal growth factor (VEGF) is one of the most relevant.^[1]^ Further, anti-VEGF therapies have been developed as oncologic treatments, and play an important role in the systemic therapy of unresectable hepatocellular carcinoma (uHCC). Systemic therapy is a good option if HCC is diagnosed as unresectable due to tumor number, size, macrovascular invasion, and metastasis. However, sorafenib and lenvatinib, which are multi-tyrosine kinase inhibitors employed in the treatment of uHCC, have been shown to cause adverse events due to VEGF inhibition, such as proteinuria.^[2–4]^

Moreover, the management of proteinuria and renal function is important in patients receiving systemic therapy for uHCC, especially regarding their long-term prognosis. Several studies have reported the effects of sorafenib on renal function, including studies on treatment of renal cell carcinoma.^[5,6]^ However, reports on the effects of lenvatinib on renal function are scarce.^[7,8]^ Furthermore, no study has directly compared the effects of sorafenib and lenvatinib on renal function in patients with uHCC.

We examined the effects of sorafenib and lenvatinib on renal function, proteinuria, and estimated glomerular filtration rate (eGFR) in patients with uHCC from historical data.

## 2 Methods

### 2.1 Patients

Patients who were administered sorafenib or lenvatinib as treatment of uHCC at the Nagasaki University Hospital from July 2009 to October 2020, were enrolled in this retrospective observational study. Of the 177 patients with uHCC, cases with first line treatment and administration of the drug longer than 4 weeks were included in the analysis. To reduce the effects of confounding factors, we used propensity scores to match uHCC patients treated with sorafenib, to uHCC patients treated with lenvatinib.

### 2.2 Definitions and proteinuria assessment

Hypertension was defined as arterial pressure measurements >140/90 mm Hg or antihypertensive drug use. Diabetes mellitus was defined as glycated hemoglobin ≥6.5% or antidiabetic drug use. We calculated the eGFR according to a previous report using the Japanese eGFR estimation formula.^[9]^

The ΔeGFR was calculated by subtracting the baseline value from the eGFR value at each measurement point and dividing it by the baseline eGFR to obtain the percentage. Cases in which ΔeGFR decreased by 10% or more were defined as renal function deterioration.

The degree of proteinuria was examined by a qualitative test using a commercially available dipstick (Uriflet S 10UB; Arkray. Inc., Kyoto, Japan). TKI dose adjustment was performed when proteinuria was detected through a dipstick test ≥2+ and urine protein–creatinine ratio (UPCR) >2.0. A report has demonstrated UPCR is strongly correlated with 24-hour proteinuria,^[10]^ with appropriate TKI dose reductions and discontinuation in cases of grade 3 proteinuria (UPCR >3.5).

### 2.3 Ethical considerations

Written consent to use medical records were obtained from each patient. The study protocol was approved by the Ethical Committee of our institution (approval number: 18052112-3) and conformed to the provisions of the 1975 Declaration of Helsinki.

### 2.4 Statistical analysis

The Wilcoxon signed-rank test and Mann–Whitney *U* test were used for statistical analysis. In the multivariate analysis, continuous variables (age, body mass index, platelet count, prothrombin time, total bilirubin, albumin, alanine aminotransferase, alpha fetoprotein, sodium, blood urea nitrogen, creatinine, and eGFR) were bisected for median or clinically meaningful values.

We performed the analysis using propensity scores. Thirteen factors were used to calculate the propensity score: age, sex, body mass index, history of diabetes mellitus, history of hypertension, history of diuretic use, etiology, performance status, Child-Pugh class, Barcelona Clinic liver cancer stage, alpha fetoprotein, creatinine, and eGFR.

The median propensity score of the sorafenib group (n = 85) was 0.3060 (0.1948–0.4435, interquartile range). The median propensity score of the lenvatinib group (n = 52) was 0.4375 (0.3425–0.6290 interquartile range). We selected 84 patients (sorafenib, n = 42; lenvatinib, n = 42) by propensity score matching (one-to-one nearest neighbor matching within a caliper of 0.2). We did not compare prognostic factors that are affected by the historical background, such as overall survival, that could not be adjusted with propensity scores. Statistical significance was defined as *P* < .05. SPSS ver. 22.0 (SPSS, Chicago, IL) was used for all analyses.

## 3 Results

### 3.1 Patient characteristics

Of the 177 patients with uHCC, 137 patients were included in the analysis. The exclusion criteria were insufficient data (n = 9), non-first line treatment systemic therapy (n = 15), and discontinuation within 4 weeks (n = 16). The baseline characteristics of the 137 patients included in this study before propensity score matching are summarized in Table 1. Participants who lacked data for each variable were excluded as insufficient data. The median observation period was 4.1 months for Sorafenib and 5.6 months for Lenvatinib. Before propensity score matching, differences could be observed between the sorafenib and lenvatinib groups regarding the Barcelona Clinic liver cancer stage, prothrombin, alpha fetoprotein, and blood urea nitrogen levels. Table 2 shows the baseline characteristics of the 84 patients selected after propensity score matching, to balance the 2 groups. After propensity score matching, the median observation period was 4.4 months for Sorafenib and 5.4 months for Lenvatinib.

**Table 1:** Characteristics of the patients.

Variables	Sorafenib (n = 85)	Lenvatinib (n = 52)	*P* value	Standardized difference
Age (yr)	70.0 (63.0–79.0)	71.0 (65.5–76.5)	.805	0.049
Sex (male/female)	66/19	44/8	.319	0.180
BMI (kg/m^2^)	22.90 (20.90–24.75)	21.70 (19.68–24.63)	.194	0.187
Diabetes mellitus (yes/no)	16/69	18/34	.037	0.363
Hypertension (yes/no)	55/30	32/20	.708	0.066
Pretreatment diuretics use (yes/no)	22/63	8/44	.149	0.262
Diuretics addition or increase during treatment (yes/no)	9/76	9/43	.258	0.194
Etiology (HBV/HCV/NBNC)	25/29/31	17/12/23	.723	0.157
Performance status 0 (yes/no)	55/30	42/10	.060	0.368
Child-Pugh class (A/B)	73/12	42/10	.428	0.137
BCLC stage (B/C)	20/65	24/28	.005	0.491
Platelet count (×10^4^/μL)	12.80 (8.70–17.33)	15.60 (10.25–20.20)	.070	0.277
PT (%)	83.0 (74.3–92.3)	88.0 (81.0–94.5)	.036	0.315
T.bil (mg/dL)	0.80 (0.60–1.10)	0.90 (0.70–1.10)	.411	0.052
Albumin (g/dL)	3.60 (3.38–3.90)	3.50 (3.20–3.85)	.386	0.125
ALT (IU/mL)	30.0 (17.0–50.0)	28.0 (17.0–41.0)	.712	0.123
AFP (ng/mL)	136.2 (11.5–1208.0)	23.0 (5.0–582.5)	.024	0.308
Sodium (mEq/l)	140.0 (139.0–141.0)	139.5 (138.0–141.0)	.171	0.225
BUN (mg/dL)	16.0 (13.0–18.0)	13.0 (11.0–17.0)	.006	0.306
Creatinine (mg/dL)	0.800 (0.720–0.920)	0.795 (0.705–0.880)	.507	0.130
eGFR (mL/min/1.73 m^2^)	72.10 (58.60–80.85)	71.90 (63.05–82.55)	.599	0.147

Data are given as the medians with interquartile range or numbers. Standardized difference is absolute value. AFP = alpha fetoprotein, ALT = alanine aminotransferase, BCLC = Barcelona Clinic liver cancer, BMI = body mass index, BUN = blood urea nitrogene, eGFR = estimated glomerular filtration rate, PT = prothrombin, T.bil = total bilirubin.

**Table 2:** Characteristics of the patients after propensity score matching.

Variables	Sorafenib (n = 42)	Lenvatinib (n = 42)	*P* value	Standardized difference
Age (yr)	70.0 (63.0–79.0)	70.0 (65.0–76.0)	.893	0.000
Sex (male/female)	33/9	34/8	.785	0.060
BMI (kg/m^2^)	21.45 (19.30–24.20)	22.85 (21.10–24.70)	.128	0.041
Diabetes mellitus (yes/no)	10/32	10/32	1.000	0.000
Hypertension (yes/no)	25/17	26/16	.823	0.049
Pretreatment diuretics use (yes/no)	11/31	7/35	.287	0.233
Diuretics addition or increase during treatment (yes/no)	4/38	7/35	.331	0.215
Etiology (HBV/HCV/NBNC)	11/14/17	14/10/18	.833	0.049
Performance status 0 (yes/no)	33/9	32/10	.794	0.057
Child-Pugh class (A/B)	35/7	35/7	1.000	0.000
BCLC stage (B/C)	15/27	17/25	.653	0.099
Platelet count (×10^4^/μL)	13.30 (9.20–18.50)	16.30 (10.60–20.50)	.168	0.220
PT (%)	83.0 (72.0–95.0)	88.5 (83.0–96.0)	.081	0.362
T.bil (mg/dL)	0.80 (0.60–1.10)	0.90 (0.70–1.00)	.967	0.153
Albumin (g/dL)	3.60 (3.20–3.90)	3.40 (3.10–3.90)	.475	0.103
ALT (IU/mL)	27.0 (17.0–50.0)	28.0 (17.0–42.0)	.914	0.052
AFP (ng/mL)	109.5 (9.0–1164.0)	39.5 (5.0–660.0)	.428	0.048
Sodium (mEq/l)	140.0 (139.0–141.0)	140.0 (138.0–141.0)	.534	0.204
BUN (mg/dL)	15.0 (13.0–17.0)	12.5 (11.0–18.0)	.171	0.182
Creatinine (mg/dL)	0.800 (0.720–0.900)	0.805 (0.710–0.890)	.875	0.167
eGFR (mL/min/1.73 m^2^)	73.30 (62.50–79.70)	70.90 (61.85–81.70)	.713	0.049

Data are given as the medians with interquartile range or numbers. Standardized difference is absolute value. AFP = alpha fetoprotein, ALT = alanine aminotransferase, BCLC = Barcelona Clinic liver cancer, BMI = body mass index, BUN = blood urea nitrogen, eGFR = estimated glomerular filtration rate, PT = prothrombin, T.bil = total bilirubin.

### 3.2 Changes in eGFR during tyrosine kinase inhibitor treatment

**Figure 1:**
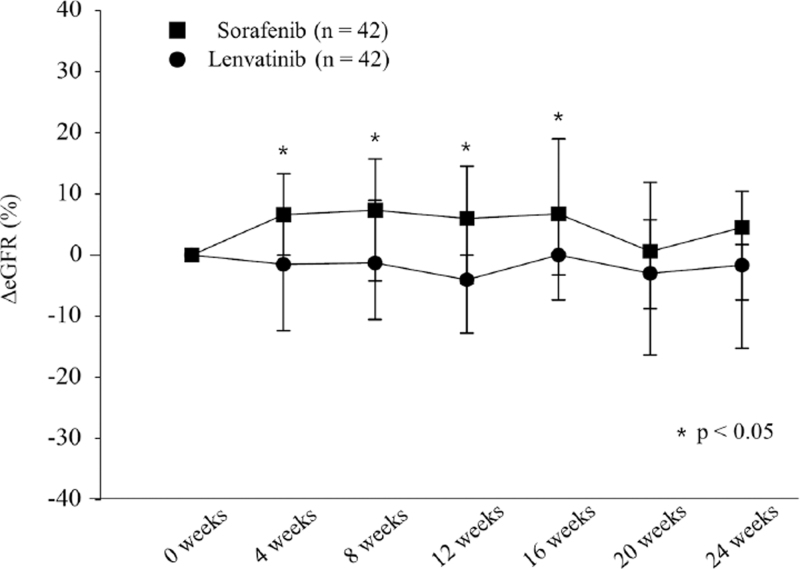
Changes in eGFR after administration of tyrosine kinase inhibitor. In the sorafenib group (black squares), the ΔeGFR was 6.6%, 7.3%, 5.9%, and 6.7% at 4, 8, 12, and 16 weeks, respectively. In the lenvatinib group (black circles), the ΔeGFR was −1.5%, −1.2%, −4.0%, and 0.0% at 4, 8, 12, and 16 weeks, respectively. The ΔeGFR differed significantly between the 2 groups at 4, 8, 12, and 16 weeks.

Figure 1 shows the changes in ΔeGFR every 4 weeks in the sorafenib and lenvatinib groups. At 4, 8, 12, and 16 weeks, ΔeGFR was significantly lower in the lenvatinib group than in the sorafenib group (*P* < .05).

### 3.3 Changes in eGFR by proteinuria

The lenvatinib group had a significantly higher frequency of proteinuria (all grades) during treatment than the sorafenib group (30.9% vs 7.1%, *P* = .005). Additionally, the frequency of grade ≥3 proteinuria was also higher in the lenvatinib group than in the sorafenib group (14.2% vs 2.4%, *P* < .049). Furthermore, the lenvatinib group had a higher rate of decrease in eGFR than the sorafenib group, and there were significantly more cases in which the renal function deteriorated ≤−10% (Table 3).

**Table 3:** Renal function-related factors associated with tyrosine kinase inhibitor treatment.

Variables	Sorafenib (n = 42)	Lenvatinib (n = 42)	*P* value
Pretreatment eGFR	73.290 (62.540–79.760)	70.935 (61.840–81.680)	.713
end of treatment eGFR	80.20 (58.60–91.80)	73.00 (51.65–81.35)	.103
Minimum ΔeGFR	−1.840 (−14.180–0.000)	−12.240 (−25.190–-1.350)	.025
ΔeGFR ≤−10% (yes/no)	13/29	23/19	.027
Proteinuria all grade (yes/no)	3/39	13/29	.005
Proteinuria grade ≥3 (yes/no)	1/41	6/36	.049

Data are given as the medians with interquartile range or numbers. eGFR = estimated glomerular filtration rate.

### 3.4 Predictors of a deterioration of eGFR

We performed a multivariate analysis of the factors predicting eGFR deterioration (ΔeGFR ≤−10%) during TKI treatment. The choice of TKI was identified as the only factor contributing to the deterioration of eGFR (lenvatinib, odds ratio 2.547 [95% CI 1.028–6.315], *P* = .043) (Table 4).

**Table 4:** Multivariable logistic regression models for deterioration of estimated glomerular filtration rate.

		Univariate analysis		Multivariate analysis	
Factor		OR (95% CI)	*P* value	OR (95% CI)	*P* value
Age	>70 yr	1.288 (0.539–3.079)	.569		
Sex	Male	0.917 (0.311–2.701)	.875		
BMI	>22.30 kg/m^2^	2.619 (1.077–6.372)	.034	2.120 (0.855–5.255)	.104
Diabetes mellitus	+	1.462 (0.533–4.007)	.461		
Hypertension	+	1.556 (0.634–3.816)	.335		
Pretreatment diuretics use	+	2.577 (0.884–7.515)	.083		
Diuretics addition or increase during treatment	+	1.720 (0.481–6.156)	.405		
Etiology	NBNC	0.668 (0.275–1.620)	.372		
Performance status	1/2	0.961 (0.342–2.704)	.940		
Child-Pugh grade	B	2.867 (0.868–9.465)	.084		
BCLC stage	C	0.943 (0.388–2.294)	.897		
Platelet count	<15.4 × 10^4^/μL	1.000 (0.421–2.373)	1.000		
PT	<87%	0.895 (0.377–2.125)	.801		
T.bil	>0.9 mg/dL	1.215 (0.511–2.886)	.659		
Albumin	<3.5 g/dL	0.636 (0.265–1.530)	.312		
ALT	>27 IU/mL	0.510 (0.212–1.226)	.132		
AFP	> 77.0 ng/mL	1.000 (0.421–2.373)	1.000		
Sodium	<140 mEq/l	0.614 (0.254–1.489)	.281		
BUN	> 14 mg/dl	0.972 (0.407–2.321)	.949		
Creatinine	>0.8 mg/dL	2.275 (0.936–5.526)	.070		
eGFR	<71.70 mL/min/1.73 m^2^	2.200 (0.911–5.316)	.080		
Thyrosine kinase inhibitor	Lenvatinib	2.700 (1.106–2.701)	.029	2.547 (1.028–6.315)	.043

AFP = alpha fetoprotein, ALT = alanine aminotransferase, BCLC = Barcelona Clinic liver cancer, BMI = body mass index, BUN = blood urea nitrogen, eGFR = estimated glomerular filtration rate, PT = prothrombin, T.bil = total bilirubin.

### 3.5 Effect of proteinuria on renal function during lenvatinib treatment

**Figure 2:**
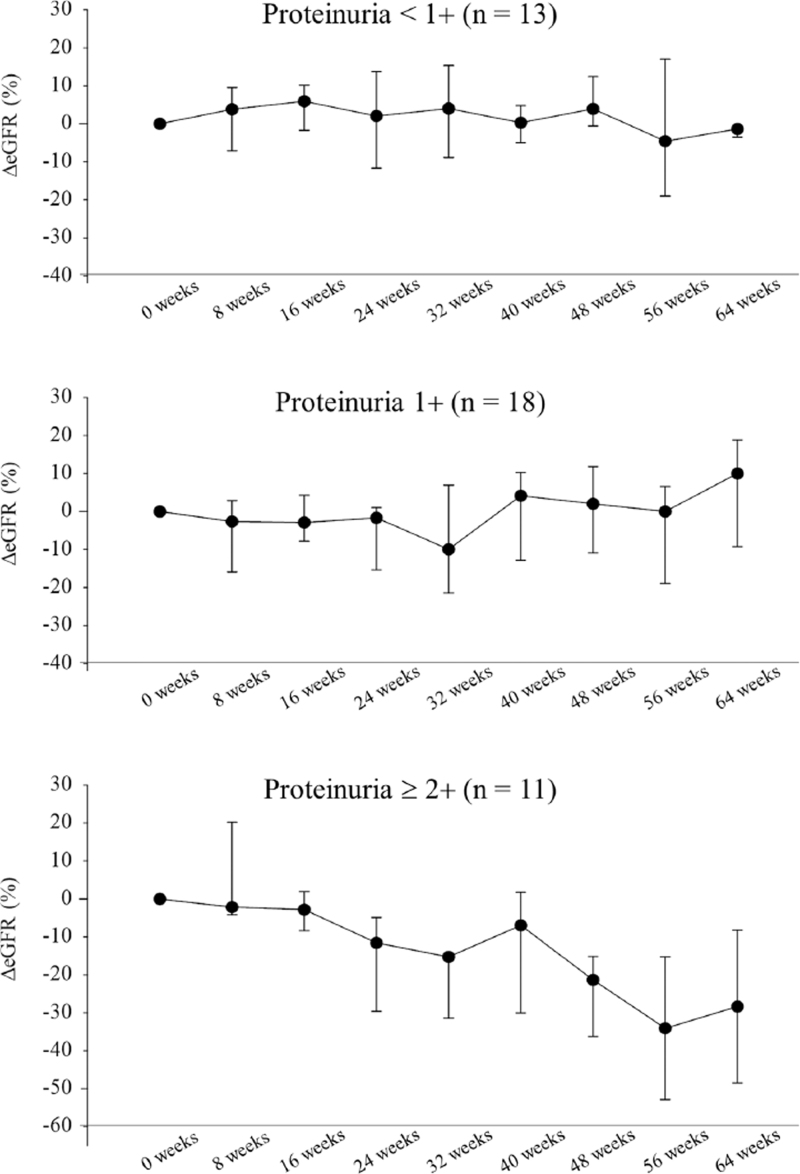
Long-term changes in eGFR every 8 weeks after administration of lenvatinib stratified by proteinuria. (A)(B) In cases of proteinuria <1+ or 1+ during lenvatinib treatment, eGFR did not decrease after 24 weeks. (C) In cases of proteinuria ≥2+ during lenvatinib treatment, eGFR decreased after 24 weeks.

Figure 2 shows the long-term changes in eGFR every 8 weeks in the lenvatinib group stratified by maximum proteinuria during treatment. In cases of proteinuria <1+ and proteinuria 1+ during lenvatinib treatment, eGFR did not suffer long-term changes, while in cases with proteinuria ≥2+, eGFR decreased in the long term.

## 4 Discussion

Tyrosine kinase inhibitors cause proteinuria through their anti-VEGF inhibitory effects.^[4]^ Given that all systemic therapies for the treatment of uHCC have an inhibitory effect on VEGF or VEGF receptors, proteinuria is an important adverse event. VEGF and VEGF receptors are essential factors in the maintenance of the glomerular barrier structure, and when these are blocked, the barrier function is disrupted and proteins leak into the urine.^[11]^ Additionally, renal function in patients with HCC has also been shown to affect prognosis.^[12,13]^ In recent years, the prognosis of HCC patients has been prolonged, and the duration of systemic therapy tends to be long due to the influence of sequential therapy.^[14]^ Therefore, the effects of systemic therapy on proteinuria and renal function are important in long-term treatments.

In our study, the frequency of proteinuria in the lenvatinib group was higher than that in the sorafenib group. Furthermore, compared to the sorafenib group, the lenvatinib group had significantly worse renal function. Moreover, a previous study comparing sorafenib and lenvatinib for thyroid cancer also reported that renal dysfunction was more frequent with lenvatinib than with sorafenib.^[7]^ Lenvatinib has a significantly higher frequency of proteinuria, but not all patients receiving lenvatinib therapy have deteriorated their renal function. Figure 2A and 2B shows that in the lenvatinib group, eGFR did not decrease in patients who have proteinuria ≤1+. However, in cases of proteinuria ≥2+, eGFR tended to deteriorate, especially in cases of long-term administration and after the 24th week of treatment (Fig. 2C). Therefore, long-term use of lenvatinib in patients with proteinuria ≥2+ carries a risk of developing an impaired renal function.

Moreover, these results were consistent with those of previous studies, that reported that proteinuria causes deterioration of renal function. Proteinuria induces tubular chemokine expression and complement activation, leading to infiltration of inflammatory cells into the interstitium and persistent fibrosis of the kidney.^[15]^ Proteinuria is believed to be the cause of renal damage, and when renal damage occurs, the burden on the residual glomerulus increases. Consequently, a vicious cycle of increased proteinuria and deterioration of renal function develops. Additionally, urinary protein has been reported to be an indicator of acceleration of renal dysfunction.^[15,16]^

Furthermore, it has been reported that the number of nephrons per kidney in the Japanese population is 600,000 to 700,000, which is less than the previously reported one million in other races.^[17]^ Further, it has been hypothesized that Japanese people may be at higher risk of renal dysfunction due to anti-VEGF therapy.

Although the importance of sequential systemic therapy in uHCC has been previously reported,^[14]^ optimal continuous treatment regimens have not been established. Proteinuria and renal function could be factors to consider drug selection since proteinuria developed during the initial treatment line has great influence on late-line treatments. Proteinuria also occurs in the atezolizumab-bevacizumab combination therapy^[18]^ currently used as the first-line treatment, which may have a great influence on the selection of the second line treatment.

One of the limitations of this study is its retrospective, single-center nature. Another limitation is that propensity score matching is performed to balance the patients baseline characteristics, but there are factors that cannot be balanced because the drugs have different historical backgrounds. Therefore, we did not analyze the patient's prognosis, which is affected by the patient's medical history and clinical situation (such as postprogression therapy), but focused on factors such as proteinuria and renal function that are not so easily affected.

Regardless of these limitations, to our knowledge, this study is the first to report the association between lenvatinib, proteinuria and deterioration of renal function in patients with uHCC. Our results indicate that lenvatinib has a greater effect on proteinuria and renal function than sorafenib, and is related to drug selection in sequential therapy for the treatment of uHCC.

## Acknowledgments

We wish to acknowledge Ms. Mai Takahira for her kind contribution to data collection.

## Author contributions

**Conceptualization:** Kazuhiko Nakao, Ryu Sasaki.

**Data curation:** Hisamitsu Miyaaki, Masafumi Haraguchi, Masanori Fukushima, Ryu Sasaki, Satoshi Miuma, Takuya Honda.

**Formal analysis:** Ryu Sasaki.

**Investigation:** Hisamitsu Miyaaki, Masafumi Haraguchi, Masanori Fukushima, Ryu Sasaki, Satoshi Miuma, Takuya Honda.

**Methodology:** Ryu Sasaki.

**Project administration:** Kazuhiko Nakao, Ryu Sasaki.

**Supervision:** Kazuhiko Nakao.

**Validation:** Kazuhiko Nakao.

**Visualization:** Ryu Sasaki.

**Writing – original draft:** Ryu Sasaki.

**Writing – review & editing:** Kazuhiko Nakao, Ryu Sasaki.
